# Psychodynamic Therapist’s Subjective Experiences With Remote Psychotherapy During the COVID-19-Pandemic—A Qualitative Study With Therapists Practicing Guided Affective Imagery, Hypnosis and Autogenous Relaxation

**DOI:** 10.3389/fpsyg.2021.777102

**Published:** 2022-01-07

**Authors:** Andrea Jesser, Johanna Muckenhuber, Bernd Lunglmayr

**Affiliations:** ^1^Department for Psychotherapy and Biopsychosocial Health, University for Continuing Education Krems, Danube University, Krems an der Donau, Austria; ^2^Independent Researcher, Vienna, Austria; ^3^Institut für Soziale Arbeit, FH Joanneum, Graz, Austria

**Keywords:** remote psychotherapy, psychotherapy, videoconferencing psychotherapy, psychotherapy via telephone, telehealth, e-mental health, pandemic, COVID-19

## Abstract

The COVID-19-pandemic brought massive changes in the provision of psychotherapy. To contain the pandemic, many therapists switched from face-to-face sessions in personal contact to remote settings. This study focused on psychodynamic therapists practicing Guided Affective Imagery, Hypnosis and Autogenous Relaxation and their subjective experiences with psychotherapy via telephone and videoconferencing during the first COVID-19 related lockdown period in March 2020 in Austria. An online survey completed by 161 therapists produced both quantitative and qualitative data with the latter being subject to a qualitative content analysis. Our research suggests that telephone and videoconferencing are considered valuable treatment formats to deliver psychodynamic psychotherapy. However, therapists’ experiences with remote psychotherapy are multifaceted and ambiguous. In particular, the findings raise questions concerning the maintenance of the therapeutic alliance, the development of the analytic process, the sensitivity to unconscious communication, and the indication for certain types of patients that still need further investigation. Our research indicates that the long-standing reticence toward remote treatments offers among psychodynamic therapists is becoming more differentiated and partially dissolves as therapists gain experiences in their use. Attitudes are becoming more open. At the same time, the way is being prepared to take a closer look at the specific processes and dynamics of remote psychotherapy and to examine them critically in future studies.

## Introduction

The COVID-19-pandemic brought massive changes in the provision of psychotherapy—and other health care services—all around the world. To avoid or reduce the risk of infection, many therapists switched from face-to-face sessions in personal contact to remote psychotherapy, i.e., psychotherapy delivered by telephone or videoconferencing.

In Austria, the government declared a first nationwide lockdown in March 2020, after the first cases were confirmed. From March 16, 2020, to April 30, 2020, a nationwide curfew restricted movement and activities, with few exceptions, such as meeting necessary basic needs of daily living, performing work tasks, and outdoor activities alone or with people from the same household. Although psychotherapy, like other medical treatments, was among the few exceptions to the complete curfew, remote treatments increased in all four psychotherapeutic orientations eligible in Austria (psychodynamic, humanistic, systemic, behavioral) (Probst et al., 2020). Until this point, psychotherapy by telephone or videoconferencing was not considered lege artis (Austrian Federal Ministry of Health, 2005) and was not covered by health insurance companies. As a result, remote psychotherapy was almost non-existent before COVID-19 (Probst et al., 2021). This situation changed in the course of the lockdown when the government expanded existing regulations (Eichenberg, 2021) and most insurance companies started covering the expenses for remote psychotherapy to the same extent as in-person psychotherapy.

Although there has been a research interest in remote psychotherapy before the pandemic, the topic has now become a “major contemporary issue” (Mitchell, 2020). Previous research supports the clinical effectiveness of remote psychotherapy among diverse diagnostic groups and on several measures of outcome (e.g., Knaevelsrud and Maercker, 2007; Carlbring et al., 2018; Norwood et al., 2018; Castro et al., 2020), with strong ratings of the therapeutic alliance by both patients and therapists (Knaevelsrud and Maercker, 2007; Germain et al., 2010; Simpson and Reid, 2014; Berger, 2017). However, most studies focus on cognitive behavioral therapy; research on psychodynamic treatments is scarce (de Bitencourt Machado et al., 2016). There have been studies positively assessing the effectiveness of online psychodynamic treatment (Andersson et al., 2012; Johansson et al., 2012; Beutel et al., 2018), but these treatments took the form of a self-therapy program based on psychoanalytic principles and involved little to no contact with the guiding therapist. There is no research assessing the effectiveness of psychodynamic treatment via telephone or videoconferencing. Gordon et al. (2015) questioned 176 therapists with a psychodynamic orientation and found that psychotherapy via videoconferencing was rated as “slightly less effective” than in-person psychotherapy on several dimensions, such as symptom reduction, exploring mental life, working on transference and countertransference, relational problems, and resistance. A second survey with 90 graduates of psychoanalytic psychotherapy revealed that the graduates highly rated the effectiveness of their own psychoanalytic psychotherapy via videoconferencing and thought therapist’s characteristics such as warmth, wisdom, empathy, and skillfulness to be far more important for the effectiveness of their treatment than whether the treatment was delivered via videoconferencing or in-person (Gordon, 2020).

The COVID-19-related switch to remote treatment formats in psychodynamic psychotherapy has received little scientific attention to date. Békés et al. (2020) surveyed 190 psychoanalytic therapists at the onset of the pandemic and inquired about their transition to psychotherapy via videoconferencing. They found that therapists felt both confident and competent in their videoconferencing sessions and that the experience of conducting session online lead to a more positive view of its effectiveness. However, there was much ambivalence regarding the quality of the therapeutic relationship and most respondents still viewed in-person treatment as more effective than remote sessions. This was confirmed by an Austrian study. A survey among all licensed Austrian psychotherapists revealed that psychodynamic therapists considered remote psychotherapy as not totally comparable to in-person psychotherapy. Interestingly, they still reported a higher comparability than behavioral therapists (Probst et al., 2020) as well as more positive experiences with remote psychotherapy than expected compared to therapists of other orientations (Humer et al., 2020).

In the psychodynamically and psychoanalytically orientated research community, remote psychotherapy has been debated on the grounds of the theoretical concepts and approaches that constitute the basis of psychodynamic practice, such as transference, countertransference, resistance, affective attachment, containment, etc. (e.g., Lindon, 1988; Mirkin, 2011; Bayles, 2012; Scharff, 2012; Dettbarn, 2013; Lemma, 2015; de Bitencourt Machado et al., 2016; Roesler, 2017; Chherawala and Gill, 2020). The relevant literature comprises theoretical contributions and single case studies of psychoanalytic sessions in which the authors are also the analysts. Tensions and a strong measure of ambiguity regarding the delivery of psychotherapy via telephone or videoconferencing are evident across the contributions. Basic questions surround the nature of the therapeutic relationship in the context of remote psychotherapy and the implications of the absence of non-verbal cues in the therapeutic interaction. While many authors express serious doubt and skepticism regarding the usefulness and effectiveness of remote psychotherapy, others consider advantages but tend to view it as second best to psychotherapy in person (Johansson et al., 2012; Scharff, 2012; Caparrotta, 2013). Still others argue that it might be a completely different medium in its own right (Scharff, 2012; Migone, 2013). Given the recent rise of remote treatment modalities, it is now a matter of urgency to further investigate their impact in a psychodynamic psychotherapy setting and to explore both therapists’ experiences of remote psychotherapy with further quantitative research and the more nuanced personal accounts of those working in the field using qualitative approaches (Mitchell, 2020).

The current study aimed to fill existing research gaps. Our main objective was to explore subjective experiences of psychodynamic therapists with remote psychotherapy during the first COVID-19 lockdown in Austria. The study was developed and conducted by members of the research advisory board of the Austrian Society for applied Depth Psychology and Psychotherapy (ÖGATAP), which is why the focus of the study is on the three psychodynamic methods represented by the ÖGATAP in Austria: Guided Affective Imagery, Hypnosis, and Autogenous Relaxation. Together, therapists of these methods account for almost a third (*N* = 841; 31.4%) of psychodynamic therapists licensed in Austria (*N* = 2,595). Most of them practice Guided Affective Imagery (*N* = 603), while Hypnosis is practiced by much fewer therapists (*N* = 154) and Autogenous Relaxation only by *N* = 57. We adopted an exploratory research approach to elicit a broad range of experiences. The aim of this publication is to shed light on these experiences and examine how they are reflected in the existing general and psychodynamically influenced discussion about remotely delivered psychotherapy. In contrast to previous single-case studies, which relied on the authors’ personal experiences in delivering remote treatment, we collected data from a representative sample of psychodynamic psychotherapists practicing Guided Affective Imagery, Hypnosis, and Autogenous Relaxation in Austria.

## Materials and Methods

### Research Design

We conducted a cross-sectional online survey from May 5 to 25, 2020—just after the lockdown was lifted by the end of April 2020. The current study was part of a larger study examining the provision of psychodynamic psychotherapy during the first COVID-19 lockdown in Austria. We received approval of the ethical review board of the ÖGATAP.

The survey was set up with LimeSurvey Professional (LimeSurvey, n.d.) and comprised 69 items focusing on changes in the provision of psychotherapy, attitudes toward remote psychotherapy, experienced changes through the remote setting, perceived limitations and difficulties as well as benefits of remote psychotherapy, and planned treatment formats after the end of the pandemic. In order to elicit therapists’ personal accounts, the survey offered several opportunities for free comments. Participants were asked to provide information about experiences they considered relevant to remote psychotherapy in general, perceived challenges of remote working, perceived advantages of remote working, their perception of the lack of physical presence in remote sessions, and their use of method-specific therapeutic interventions (i.e., guided imageries, hypnotic trances, or autogenic relaxations). They could also choose to share further information via a free comment box at the end of the survey. Quantitative results have already been published by Jesser et al. (2021); the results of the qualitative analysis of all free text comments will be presented in this paper.

### Participants

Austria has a long tradition of psychotherapy going back to the 1920s, and a wide range of established psychotherapy methods, which can be classified into four orientations (psychodynamic, humanistic, systemic, behavioral). Currently, 2,595 psychodynamic therapists from 12 different psychodynamic methods are officially registered (Austrian Federal Ministry of Social Affairs, Health, Care, and Consumer Protection, 2021). Psychodynamic methods include (1) Analytical Psychotherapy, (2) Group Psychoanalysis, (3) Individual Psychology, (4) Psychoanalysis/Psychoanalytical Psychotherapy, (5) Psychoanalytically oriented Psychotherapy, (6) Autogenous Relaxation, (7) Daseinsanalysis, (8) Dynamic Group Psychotherapy, (9) Hypnosis, (10) Guided Affective Imagery, (11) Concentrative Movement Therapy, and (12) Transactional Analysis (Heidegger, 2017).

The ÖGATAP offers basic and advanced training for the methods Guided Affective Imagery, Hypnosis and Autogenous Relaxation and most therapists maintain their membership even after finishing their training. A link to the online survey was sent by the Society’s board to all registered members who (1) had either completed their psychotherapy training or were treating patients under supervision in the last part of their training, (2) were practicing one of the three psychotherapy methods and (3) were currently working with patients, whether adults or children and adolescents (*N* = 687). Participation was voluntary, without incentives. Participants had to agree to the data protection declaration to start the survey (electronic informed consent). The principles outlined in the Declaration of Helsinki were followed.

### Data Analysis

Qualitative data was subjected to a directed approach to qualitative content analysis (Hsieh and Shannon, 2005) using the software Atlas.ti (Atlas.ti, 2018). Two authors (AJ and BL) conducted the analysis. They are both trained psychotherapists practicing Guided Affective Imagery, members of the ÖGATAP‘s research advisory board and active in psychotherapy research. As a post-doctoral sociologist, AJ has many years of experience in qualitative research. BL is a humanities scholar and is currently working on his Ph.D. on the role of the body in psychotherapy. Their background and understanding of psychodynamic theory were valuable for the analysis process, e.g., to contextualize condensed theoretical statements. However, it was necessary to reflected on their own experiences and attitudes toward remote therapy in regular research sessions to remain open to the diversity of views contained in the data.

In sum, 571 free text comments were received: 88 about relevant experiences in remote psychotherapy, 125 about challenges of remote working, 103 about advantages of remote working, 140 about experiences regarding the lack of physical presence, 61 concerning the use of therapeutic interventions, and 54 in the final free text comment box. 151 (93.8%) therapists answered at least one of the six open questions, 143 (88.8%) therapists answered more than one open question and 19 (11.8%) therapists answered all open questions. Only 10 (6.2%) therapists answered none of the open questions. A part of the comments was brief (e.g., statements of clarification, short comments about a range of issues), but many of them comprised more detailed accounts of personal experience and reflection. The shortest comment comprised three words, the longest comment comprised 178 words. In sum, all comments comprised 11.433 words.

To familiarize ourselves with the material and obtain a sense of the whole, two authors (AJ and BL) first read all the comments. This revealed the multidimensional nature of respondents’ experiences, and it became clear that using the topic of the respective question as a deductive coding scheme, i.e., “challenges,” “benefits,” etc., would prevent us from capturing the complexity of the data. Therefore, a review of the relevant literature was used to identify several key concepts as initial coding categories: therapeutic relationship, therapeutic process, content of sessions, perception of transference, and countertransference, lack of physical presence, and therapeutic interventions. In a joint session, AJ and BL determined category definitions and coding rules for each deductive category, thereby developing an initial coding scheme. They started coding together, which helped to revise and clarify category definitions and adapt coding rules. It was also necessary to add categories to the coding scheme inductively, e.g., patient-specific observations, therapists’ experiences, and characteristics of the setting. When further coding did not reveal new categories and category definitions and coding rules were precise enough to unanimously code the comments, coding was continued separately by subsuming each answer to one or more categories of the coding scheme (some text passages were allocated to several categories if different aspects were addressed). It should be noted that initial categories were broad and text passages were subsumed without further definition of sub-categories.

**FIGURE 1:**
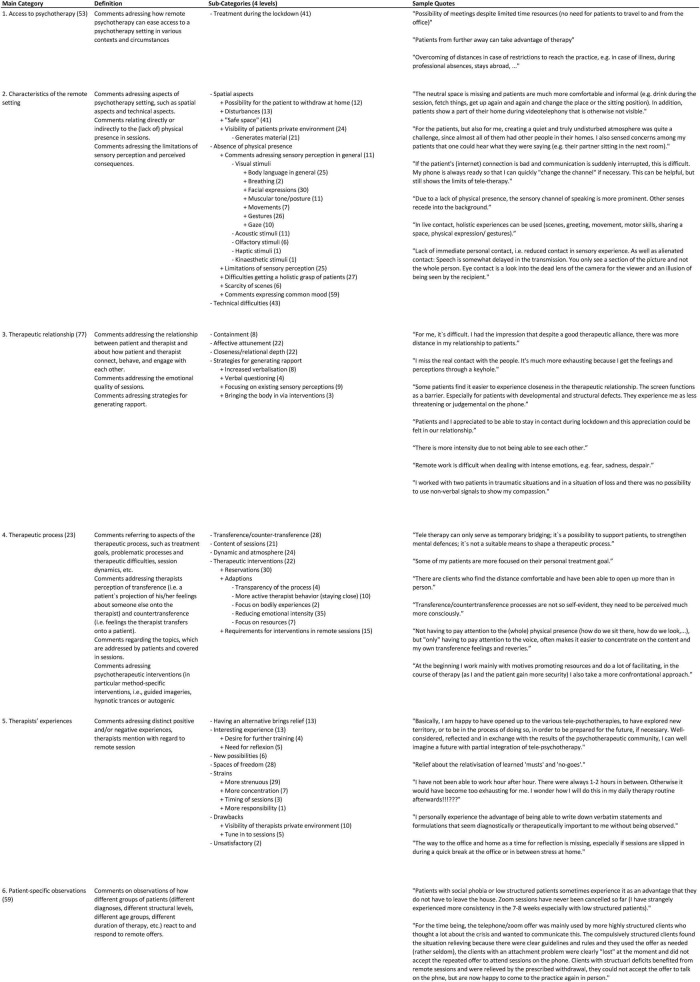
Provides an overview of the structure of categories. Column 1 captures the main categories by which the chapters of the paper are organized. Column 2 provides a category definition. For larger categories, definitions of sub-categories at level 2 are included as well. Column 3 lists the associated sub-categories. The indentation indicates the level of the sub-category. Sub-sub-categories are indented accordingly. Column 4 contains a selection of quotations for the respective main category. Attention has been paid to illustrate different aspects of the main category in the quotations. The numbers in brackets indicate the number of allocated text passages. In some cases, the main categories function as an umbrella for sub-categories and do not themselves contain quotations. In other cases, more general comments have been subsumed under the main category and more detailed comments addressing specific aspects of the main category have been subsumed in different sub-categories.

In a next step, all comments in each main category were screened word by word and sub-categories were developed inductively. AJ and BL worked separately and iteratively discussed their progress, i.e., new sub-categories that emerged from the data or comments they found difficult to allocate to a sub-category. This was an intense process of structuring and making sense of the data, which was both systematic and complex and involved a back-and-forth movement between the whole set of categories and parts of the text. It was necessary to rethink the initial coding scheme, identify relations between, and build a hierarchy of, categories. Some of the initial categories remained main categories, such as “therapeutic alliance.” Others became part of a main category—for example, this was the case for the category “content of sessions,” which became a sub-category of “therapeutic process.” The entire process was accompanied by extensive memo writing that helped sort the data and increase the level of abstraction step-by-step (Lempert, 2007). The result was an elaborated system of categories and sub-categories on various levels of abstraction. The final structure of the categories is presented in Figure 1, which displays the main categories and category definitions, a list of the associated sub-categories on four levels of abstraction, and a selection of quotations for the respective main category. Attention has been paid to illustrate different aspects of the main category in the quotations. The chapters in the results section correspond to the main categories of the analysis and address the major themes and areas of experience reported by the therapists, who participated in the study. We refrained from presenting our results based on frequencies because we found it problematic to weight the comments. Instead, we wanted to show the range of reported experiences and use an exploratory approach to provide starting points for further research.

## Results

### Sample Description

In total, *N* = 161 psychotherapists completed the online survey; this amounts to a respondence rate of 23.4%. A comparison of the distribution of their psychodynamic methods with the distribution of the three investigated psychodynamic methods in the official Austrian list of psychotherapists showed that the distribution was representative (% in the study sample vs. % according to the Austrian list of psychotherapists): Guided Affective Imagery 72.9 vs. 74.1%, Hypnosis 17.7 vs. 18.9%, and Autogenous Relaxation 4.4 vs. 7%. The sample was also representative for gender (81.4% female psychodynamic psychotherapists in the survey vs. 78.1% female psychodynamic psychotherapists of the three investigated psychodynamic methods in the Austrian list of psychotherapists in March 2020). 14.3% were younger than 40, the majority of participants (67.1%) were between 41 and 60 years old, and 18.6% were older than 60. In terms of professional experience, 28% worked as a therapist for less than 5 years (this category includes therapists in training under supervision), 24.8% between 5 and 10 years, just as many between 10 and 20 years, and 22.4% over 20 years. Sociodemographic characteristics are summarized in Table 1.

**TABLE 1:** Sociodemographic characteristics of the sample.

Characteristics	*n*	%
**Gender**		
Female	131	81.4
Male	28	17.4
Others	2	1.2
**Age**		
≤40	23	14.3
41–50	62	38.5
51–60	46	28.6
>60	30	18.6
**Years in profession**		
≤5	45	28.0
5.1–10	40	24.8
10.1–20	40	24.8
>20	36	22.4
**Psychodynamic method^a^**		
Guided Affective Imagery	132	72.9
Autogenous relaxation	8	4.4
Hypnosis	32	17.7
Others	9	5.0

*^a^Multiple responses were possible.*

### Main Category 1: Access to Psychotherapy

Respondents described how remote psychotherapy could ease the access to or enable the maintenance of an ongoing psychotherapy. They referred to several contexts, including the possibility to continue treatment without the risk of infection during the pandemic. Additionally, they mentioned that remote sessions enabled therapy for patients who had limited time (work, children, etc.), who moved or lived remotely, who had care responsibilities at home, and who suffered from illnesses or physical disabilities.

### Main Category 2: Characteristics of the Remote Setting

#### Sub-Category 2.1: Spatial Aspects

Remote sessions take place in patients’ private rooms. Therapists experienced this as an additional channel of perception and an opportunity to get a more differentiated picture of patients. They mentioned how they could see patients in their familiar environment, witness some of their living circumstances and gather scenic impressions, e.g., how patients positioned themselves in front of the camera or how they interacted with family members. At the same time, therapists perceived patients to be distracted and also reported how glimpses into private spaces distracted themselves.

Therapists commented that for some patients it was difficult to establish a safe space at home. If patients lived with someone, they had to arrange a room and a time for a session to take place. Therapists also reported that some patients were concerned that someone—in their home or in the therapist’s home—might be listening in.

Therapists pointed out that the remote setting prevented them from providing the holding experience that is conveyed by sharing a room. They also described that the remote setting lacked the “transitional space” of the office, as patients could not “leave behind” stressful content of the session in the office. This content remained present at home—and patients were left alone with it. Additionally, there was no way from the office to the patient’s home, which could have helped to gain distance from the events.

#### Sub-Category 2.2: Absence of Physical Presence in Remote Psychotherapy

Therapists described that the absence of physical presence in remote psychotherapy had a comprehensive effect on the therapeutic experience. They pointed out the severe limitation of sensory perception, in particular, the loss of body language, but also a lack of other visual, acoustic, olfactory, haptic, and kinesthetic stimuli. Disturbances in image or sound transmission and the impossibility of having direct eye contact could make the virtual contact feel artificial.

Respondents further mentioned difficulties getting a holistic grasp of patients by using all their senses. They missed experiencing how patients enter the room, how it feels to shake their hand, how they smell, how they move and let their gaze sweep through the room, how they hold or avoid eye contact, how they lean in or away from the therapist, how they move their hands or tap their feet unconsciously while speaking, how they breath rapidly or hold their breath, how their muscles tighten or relax, how it feels to be in a room or look at a picture together, etc. This goes hand in hand with a scarcity of scenes that usually unfold in the therapeutic encounter. Respondents noted that the remote setting can be more easily instrumentalized for enactments, e.g., to hide something. Given the restricted nature of the visual encounter, therapists expressed concerns about missing warning signs, e.g., when a patient with anorexia is losing weight.

### Main Category 3: Therapeutic Relationship

There was a strong measure of ambiguity regarding the quality of the therapeutic relationship in remote psychotherapy. Some respondents expressed the opinion that the relationship was inferior in the context of the remote setting. They used adjectives such as inauthentic, non-committal, distanced, or impersonal to describe their relational experiences and elaborated that they found it hard to connect relationally with patients, to feel close to them and to stay attuned. They related this to various and interrelated factors.

• Not sharing a physical space with patients and the actual spatial distance between therapist and patient were experienced as a distance in the therapeutic relationship. Therapists described the screen as a barrier between them and the patient and a feeling of being separated.
• Limitations regarding sensory perception evoked a lack of confidence in therapists. They felt unsure about their ability to sense the momentary emotional state of their patients or assess how interpretations were received. Therapists felt constrained in their capacity to communicate empathy, especially when facing strong emotions such as fear, sadness, anger, or despair.
• Technical difficulties and the unreliability of virtual contact left therapists feeling constrained in maintaining a holding therapeutic space that is able to facilitate intimacy with their patients.

Other respondents perceived the development of a deeper therapeutic relationship. Their statements indicated that the effort to maintain continuity of sessions during the crisis through remote contact led to a new appreciation in the relationship and that patients felt contained and supported as a result. Therapists described how the external situation connected them with patients and it strengthened the relationship to go through a difficult time together.

Regardless of the crisis, therapists claimed that the remote setting generated closeness in the therapeutic relationship. The proximity of the face on the screen and the other person’s voice at the ear created a feeling of intimacy and enabled therapists to remain attuned and connected. Facial expressions could be observed more closely than in the face-to-face setting, which facilitated attunement to patients’ emotional responses and—on the part of the therapists—was used to convey the expression of empathy and warmth. In psychotherapy via telephone, respondents described a “sharpening of hearing.” The lack of other stimuli led to a refined perception of the tone of voice and speech melody, which enabled them to stay attuned. Therapists also observed how they compensated for limitations in non-verbal communication by giving more verbal responses (hmm, uh-huh) or using empathic words, by speaking in a richer voice or using more explicit intonation.

Moreover, therapists pointed out that the physical distance could convey a degree of protection and anonymity that was sometimes beneficial to establishing a more intimate connection. They observed that some patients felt less threatened and experienced the therapist as less judgmental, which resulted in greater disinhibition and openness. Respondents particularly referred to patients, who are uncomfortable with emotional closeness and schizoid patients.

### Main Category 4: Therapeutic Process

Experiences regarding the therapeutic process in remote psychotherapy were multifaceted as well. Therapists mentioned that processes were halted or obstructed with some patients. For them, the remote setting provided little room for psychic development. Therapists stated that they could only work in a supportive way, if at all, and refrained from using therapeutic interventions. They explained this as their insecurity about being able to contain potential ruptures and create a safe therapeutic space for working on deeper dynamics and stressful issues. In addition, they referred to their lack of experience and training.

Respondents also perceived that it was more difficult for patients to engage in deeper processes. They noticed a tendency among patients to talk and avoid silence as well as topics relevant to therapy. At the same time, they observed, how they asked more questions and talked more instead of pacing or resonating with what could be perceived non-verbally. Focusing on the conversational content of a session encouraged rationalization, which made respondents feel that sessions remained “on the surface.”

Therapists also stated that they found it difficult to engage in transference. Without the usual wealth of sensory perceptions, they were afraid to miss or misinterpret transference reactions. Respondents described problems feeling and understanding their countertransference and using it to inform interventions specific to the therapeutic method (i.e., guided imageries, hypnotic trances, or autogenic relaxations). They argued that discerning transference processes required much more attention in the remote setting.

In other cases, therapists described how processes could be advanced or even deepened in remote sessions. They observed that patients were more focused on themselves, on topics, and on treatment goals. They related this to the limitation of sensory perception, which facilitated concentration on the conversation and verbalization of affects that might have been conveyed through non-verbal communication in a face-to-face setting. The explicit specification and interactive attunement of affect and experience was described as beneficial for the therapeutic process.

Moreover, the remote setting was found helpful for engaging in transference. Being alone in their own private space, therapists felt that they were better able to let their countertransference unfold. They also noticed that transference patterns became more obvious in remote psychotherapy and psychodynamic themes were revealed or accentuated within the therapeutic relationship, e.g., a patient’s anxiety of separation. Remote sessions offered the possibility to process such emergent feelings. Furthermore, therapists observed that the physical distance allowed patients to experience their achievements in therapy more independently, e.g., the development of a secure attachment or a positive self-experience, which facilitated further internalization.

Therapists also mentioned that patients were less inhibited in the remote setting and disclosed difficult topics and feelings that had not previously been brought up in the face-to-face setting. In particular, they found that shame-related issues were more readily addressed in remote sessions, which at times made them experience the therapeutic process more intensely than in-person sessions.

While therapists emphasized how they had been oriented toward stabilization when starting to deliver sessions remotely, they became confident in working more confrontationally as they and their patients gained familiarity with the respective medium. When working with therapeutic interventions, they focused on resources and adapted the intervention to reduce the emotional intensity of the affect experienced. Moreover, they made sure to discuss the process of the intervention in more detail with the patient in advance and paid attention to the patient’s environment—where the patient sat, if someone was there in case of an emergency—and whether there was an undisturbed, confidential atmosphere. Therapists further described how they tried to be “closer to the patient” during interventions, e.g., by adopting a more active behavior than in the face-to-face setting and exploring more closely what patients were experiencing. They also tried to bring in the absent body by asking for body perceptions and using interventions focusing on the body.

### Main Category 5: Therapists’ Experiences With Remote Psychotherapy

Therapists reported both positive and negative experiences with remote psychotherapy. Amongst the positive experiences was a relief to have an alternative way to offer treatment during the crisis. Amongst the negative experiences were feelings of anger and frustration regarding technical problems and the limitations of the remote setting. We summarized further statements in different categories.

• Therapeutic opportunities: Therapists mentioned how their initial skepticism regarding remote psychotherapy was reduced through practicing it during the lockdown. They were surprised that psychotherapy could be adapted to a remote setting and described the experience as “interesting,” “enriching,” and “empowering.” Therapists argued that the non-standardized setting created a space to explore new possibilities outside of routine procedures and to find their own ways and approaches. They found it valuable to reflect their work in intervision and supervision as well as exchange their experiences with the psychotherapeutic community.
• Spaces of freedom: Working remotely made therapists feel more independent in terms of location. They could hold sessions from home or continue to see patients whilst away from home themselves. Working from home saved time spent getting to the office and back, which facilitated a better coordination of professional appointments and private commitments and reduced stress. Being invisible during sessions via telephone allowed therapists to take notes unobserved, to dress, and position themselves more comfortably, to drink, etc.
• Strains: Therapists described the remote setting as more strenuous and tiresome. Compensating reduced sensory perceptions with attention to acoustic and/or visual impressions required more concentration. Respondents mentioned that it could be difficult to work for several hours consecutively. Extended breaks between sessions could lead to sessions being more spread out over the day or the week.
• Drawbacks of working from home: Establishing a quiet and undisturbed atmosphere could be difficult for therapists as well. They too showed something of their private space, which for some therapists created an unwanted closeness. They preferred to offer remote psychotherapy from the office or made arrangements to protect their private space, for example, by setting up a neutral background for the video call. Working from home also entailed less time to attune themselves to sessions, as sessions were scheduled in between other appointments and the way to the office and home was missing as a time for reflection.

### Main Category 6: Patient-Specific Observations

Respondents stated that it was easier to switch to a remote setting if there had been prior contact with a patient. In this case, they could draw on their experiences and observations of, e.g., a patient’s habitual bodily movements or reactions. The remote setting was considered unsuitable for first consultations. Respondents also observed that adolescents opened up more easily in remote psychotherapy. With children, on the other hand, remote work was considered as hardly possible.

Therapists stated that patients responded very differently to the remote therapy offer. While some patients felt contained and supported by the continuation of their therapy during the lockdown, others experienced feelings of separateness and alienation. The absence of physical contact caused anxiety and some patients withdrew and closed themselves off from the therapist because they felt left alone. While some patients wanted to switch back to in-person sessions as soon as possible, others settled in too much in the distance.

Respondents noticed that patients on higher levels of personality structure could better use the remote setting to communicate their thoughts about the crisis and to reflect on their experiences and actions based on their symbolic function and reflective-integrative capacity. On the contrary, patients with developmental and structural deficits had difficulty with the remote setting and generally accepted it less. They reacted to the spatial separation with anxiety and their symptoms intensified. However, regular appointments provided structure and the spatial distance was helpful in cases where therapists were experienced as too intrusive or threatening in the face-to-face setting. The different levels of personality structure (high, medium, low) are derived from a psychodynamic model used to organize disorders along a structural continuum of severity. High refers to a neurotic level, i.e., the healthiest level of personality organization, describing people with an intact reality check, a consistent view of self and others, and mature defense mechanisms. Low refers to a borderline level, i.e., a low level of personality organization that describes people with difficulties in reality testing, an inconsistent sense of the self and others, and primitive defense mechanisms. Medium refers to the level at the transition between the neurotic and borderline level (Ermann, 2007).

With regard to specific mental disorders respondents observed that the remote setting worked well for patients with anxiety disorders, who appeared more self-confident in their home. However, the remote setting could also contribute to making problems less visible. Respondents considered it difficult to work with trauma-related material because they felt limited in their ability to contain the patient. They perceived that patients who suffered from depressive disorder were more hesitant to switch to remote psychotherapy and tended to withdraw over the course of the first lockdown. When therapists returned to an in-person setting after the lockdown, some of these patients never resumed their sessions.

## Discussion

To the best of our knowledge this is one of the first studies investigating remote treatment formats in psychodynamic psychotherapy during the COVID-19 pandemic and one of the few studies using a qualitative approach to assess therapists’ subjective experiences with psychotherapy via telephone and videoconferencing. The findings suggest that therapists’ experiences are multifaceted and ambiguous.

Respondents emphasized that remote psychotherapy enabled them to provide psychotherapy during the pandemic and facilitates access to psychotherapy in general. Their arguments are reflected in the literature on remote psychotherapy (e.g., Bee et al., 2008; Brenes et al., 2011; de Bitencourt Machado et al., 2016; Connolly et al., 2020; Simpson et al., 2020; Stoll et al., 2020; Weightman, 2020) and reveal a thoughtful consideration of the possible uses of remote settings during and independently of the pandemic, as well as a fundamentally open stance toward remote therapy. Therapists also mentioned personal advantages, such as spatial and temporal flexibility and described a sense of pioneering. Many were forced by the pandemic to offer remote therapy for the first time and experienced unexpected opportunities to shape the therapeutic encounter. On the other hand, the remote setting was experienced as more strenuous. This has also been found in other studies (Békés et al., 2020; McBeath et al., 2020). Our research provides insight into the underlying reasons. Handling technical disruptions and compensating for missing sensory perception was described as tiring. Working from home and mixing personal and professional spheres meant that there was little time to tune into or reflect on sessions.

Simpson et al. (2020) discussed the “democratizing effect” that results from patients “being situated in their own ‘territory.”’ Based on qualitative interviews with integrative psychotherapists, Mitchell suggested that remote psychotherapy might cause a shift in the power balance between patient and therapist as both therapists and patients share their personal environment (Mitchell, 2020). Therapists in our sample confirmed that remote therapy enabled patients to co-create the setting, which facilitated involvement in therapy. They found that witnessing patients in their private home environment provided valuable scenic information. However, they also pointed out that they as well as their patients had difficulties establishing an undisturbed atmosphere at home and that sometimes, insights into private life created an unwanted closeness. Furthermore, therapists addressed the lack of the “transitional space” of the office where patients can leave burdensome content behind.

In the literature, the virtual space is conceptualized as “transitional space” as well. It is argued that the virtual space resembles a sphere of play in which patients can be creative and experiment with their developing identities. The therapist as the significant other is there and simultaneously not there, which creates a protected space for the patient, facilitating psychic development (Dettbarn, 2013; Lemma, 2015; Roesler, 2017; Chherawala and Gill, 2020). In our data, we found evidence that this perception was shared at least by a part of our respondents, who argued that the remote setting enabled patients to experience accomplishments in therapy more independently and thus internalize accomplishments further. Considering different accounts in our data as well as in the literature, it would be of special importance to investigate and understand more profoundly the effect of different spaces, real, and virtual, on psychodynamic therapies and to expand research on the chances and limitations of the virtual encounter for psychological maturation.

The therapeutic relationship is a fundamental aspect of psychodynamic psychotherapy (Johansson et al., 2012). In our study, therapists addressed both a distance and a closeness in the relationship. They mentioned that the spatial separation evoked a sense of separateness both in the patients and in themselves, and that technical disruptions made contact feel artificial. Because they could not draw from the wealth of sensory perceptions, therapists found it hard to stay attuned and to provide a stable base for containment and safety. They mentioned difficulties grasping and understanding transference processes. However, therapists also claimed that it was possible to compensate for missing non-verbal communication by focusing on other channels of perception, and thereby remain attuned.

The ambiguity of these findings is echoed in the quantitative results of Békés et al. (2020), showing that half of the surveyed therapists felt as emotionally connected in remote sessions as in the face-to-face setting, while the other half of the respondents felt less connected. It is also reflected in controversial theoretical contributions as well as in observations from single-case studies. Bayles (2012) pointed out how therapeutic action is fundamentally grounded in implicit and procedural non-verbal communication, which she found to be dampened in remote psychotherapy. Brenes et al. (2011) also stressed that affective attunement might be difficult without full access to non-verbal communication. Supporting her argument, Roesler (2017) emphasized that emotional security is conveyed mainly through non-verbal cues and the loss and distortion of non-verbal elements might cause misunderstandings, insecurities and ruptures in the relationship and ultimately endanger its holding function. He argued further that the absence of reality input might intensify an idealization of the therapist. As one of the transformative qualities of the therapeutic process is the experience of a new emotional relationship with a significant other, psychological maturation might become obstructed in a virtual therapeutic space without an authentic real-life relationship. Other authors took a more optimistic stance, pointing out that unconscious elements of communication might be presented differently in remote settings (de Bitencourt Machado et al., 2016) and that it is not the physical proximity that is necessarily decisive for feeling close, but instead that it is a “true encounter” (Manguel, 2019). Similar to the respondents in our study, Scharff (2012) emphasized that unconscious communication can occur on various channels of perception. Referring to her own experience, she observed that even without the other person being physically present, the body “joins in the conversation.” Given the paucity of empirical studies, further research, and particularly observational studies, are needed to understand more profoundly the “complex mixture of proximity and distance, of presence and absence, of reality and fantasy” (Roesler, 2017) that characterizes remote interaction and produces new forms of social relationships that differ significantly from face-to-face interactions. In particular, it is necessary to explore how these relationships enable the patient to form a secure attachment and use that as a lever for change (Chherawala and Gill, 2020).

Interestingly, some therapists in our study described a more intense feeling of being separated from their patients than others. Indeed, it has been pointed out that some therapists may feel less comfortable outside the usual setting (Mirkin, 2011; Scharff, 2012; Zeavin, 2020). On the other hand, therapists observed that patients also accepted the remote setting differently depending on their level of personality structure and psychiatric disorder (Jesser et al., 2021). Previous research provides limited and inconclusive evidence regarding the advantages of remote psychotherapy to certain types of patients. Consistent with our findings, there is some indication that remote psychotherapy might be a less-threatening treatment option for patients with a high degree of social anxiety (de Bitencourt Machado et al., 2016; Simpson et al., 2020). While other studies concluded that remote psychotherapy might be beneficial for patients with posttraumatic stress disorder, who might feel less overwhelmed by the immediate presence of the therapist in the safety of their home environment (Roesler, 2017; Simpson et al., 2020) and more free to reveal split-off parts of the self and overcome fears regarding intimacy (Scharff, 2012), our findings suggest that trauma-related material can be difficult to contain in a remote setting. As existing studies on the efficacy of remote therapy for different groups of patients are often compromised by methodological problems (Chakrabarti, 2015), further research is needed on this question. It is also necessary to understand how and why these patients do or do not benefit from remote psychotherapy and how they relate to the therapist differently in the remote encounter. Qualitative approaches focusing on patients’ perspectives could be beneficial to shed light on these questions.

Concerning the therapeutic process, therapists described different experiences as well. Some respondents framed the remote setting as unsuitable for psychic development. They felt that they could only work in a supportive way because they could not contain difficult emotions. For the same reason, they refrained from using interventions typical for their therapeutic method. They experienced sessions to be more superficial and described a tendency to talk and avoid silences in patients and in themselves. These results are consistent with those of Huscsava et al. (2020), who suggested that therapeutic processes become more superficial in remote psychotherapy. However, in the psychoanalytically oriented debate, a number of authors pointed out that these dynamics may be understood as defenses on the parts of both patient and therapist and need to be analyzed and addressed as such in therapy (Scharff, 2012; Migone, 2013). Dettbarn (2013) pointed out that it can be difficult to understand silence in remote psychotherapy because it needs to be clarified whether it is an active silence on the part of the conversation partner or a disturbance in the connection. Lindon (1988) observed that the urge to talk decreases as therapists gain experience and feel more at ease in the new setting.

Other respondents described how therapeutic processes could be advanced and deepened in remote sessions because patients were more focused and brought new topics into therapy. Existing literature also pointed to the disinhibition effect in remote therapy (Mirkin, 2011; Migone, 2013; Stoll et al., 2020). It was associated with patients having more control over the setting and feeling less threatened by the therapist (Simpson et al., 2020). Mitchell (2020) argued that automatic psychological defenses that are generated by being in a room together are lowered in the remote encounter. Roesler (2017) added that patients need to fill information gaps that are caused by limitations of sensory perception with intensified self-disclosure.

Furthermore, respondents in our study experienced that the remote setting made certain psychodynamic themes more visible. They observed that transference patterns became more obvious and it was easier for them to let their countertransference unfold. These accounts are supported by Migone (2013), who argued that transference manifestations might differ depending on the context in which the patient-therapist interaction takes place and that remote psychotherapy brings to light new transference configurations. In addition, therapists observed that losses in non-verbal communication necessitated increased verbalization and explication of affects, which was beneficial for creating a deepened sense of mutual understanding. This was also reported by Mitchell’s (2020) interview partners.

The qualitative analysis provided a perspective on therapists’ multi-layered experiences with remote psychotherapy and revealed their reflective and largely open attitude. The quantitative results confirm that most therapists are willing to switch to remote treatment formats, if necessary (Jesser et al., 2021). However, many therapists reported negative effects of remote psychotherapy and prefer seeing their patients in-person. Whether this is due to a lack of knowledge and experience or difficulties in delivering psychodynamic psychotherapy in a remote setting requires further investigation.

### Limitations and Strengths

This study has several limitations. First, the study was limited to the three methods Guided Affective Imagery, Hypnosis and Autogenous Relaxation, which represent only a part of the psychodynamic methods accredited in Austria. Including all 12 psychodynamic methods in the survey would have provided more comprehensive evidence as well as an assessment, whether the specifics of the investigated therapeutic methods have an impact on the perception of remote sessions. We assume that they are comparable to other psychodynamic methods in sessions with an emphasis on conversation. However, with regard to the use of method-specific interventions (i.e., guided imageries, hypnotic trances, or autogenic relaxations), our data indicate particularities that could be further investigated in follow-up studies. Second, it was conducted online, which might have caused higher participation of therapists with a preference for psychotherapy via videoconferencing. Third, we assessed only therapist’s experiences—patients accounts might differ. Fourth, time is a relevant factor when addressing experiences with remote psychotherapy during the COVID-19 pandemic. We conducted our survey at the beginning of the pandemic, after an initial lockdown of nearly 7 weeks in which many therapists switched to remote psychotherapy. It is possible that a different picture would emerge now, after a longer experience with remote psychotherapy.

A major strength of this study is that it yields findings about therapists’ perspectives on remote psychodynamic psychotherapy. As has been shown, research on remote psychodynamic treatments consists of single case studies and theoretical contributions. Both strands of research provide valuable insights that we were able to integrate in our study. On the one hand, empirical observations from single case studies were found to be reflected in a broader population; on the other hand, theoretical findings could be used to substantiate empirical observations. Another strength of the study is its focus on therapists’ personal accounts. Although qualitative interviews would have added depth to some of the issues raised in the survey, many free-text comments included details that opened up a nuanced picture of personal experiences and revealed relevant themes for further study.

### Lines of Future Research

In summary, we see several possible areas for future research on remote psychodynamic therapies arising from the main themes we have identified in our research:

• Following up on category 2: Explore the role of the body in remote therapy, how the body can be integrated and what may be lost in remote sessions. Gain an understanding of how spatial distance affects the therapeutic situation, whether chances for psychological maturation are created or patients feel left alone in their own (virtual) space. These questions need further refinement. Qualitative research designs could be employed here.
• Following up on category 3: Understand more profoundly how emotional closeness and relational depth emerge as an interactional accomplishment in remote sessions. A conversation analytical perspective, as applied by Knol et al. (2020) to investigate how silence attains interactional meaning in a psychotherapy context, might be helpful to investigate this phenomenon. The analysis could be differentiated by looking at different patient groups (diagnoses, levels of personality structure, age, etc.). Otherwise, a triangulation of qualitative interviews with patients and therapists about their experience of the therapeutic relationship in remote sessions could provide insights.
• Following up on category 4: Shed light on the development of the psychodynamic process, including transference-countertransference, in remote therapy. This question could be investigated both quantitatively and qualitatively within the framework of psychotherapy process research.
• Following up on category 6: Investigate the effectiveness of remote psychodynamic psychotherapy, the advantages for certain types of patients as well as contra-indications for remote treatment. These questions require a quantitative approach.

## Conclusion

Today, remote psychotherapy is a “modern reality” (de Bitencourt Machado et al., 2016)—even more so since the outbreak of the COVID-19 pandemic, which facilitated a rapid increase in remote treatment formats all around the world. Our study highlighted that psychodynamic therapists in Austria, who practice Guided Affective Imagery, Hypnosis, and Autogenous Relaxation, consider benefits and in general adopt an open attitude toward remote psychotherapy. However, they addressed a number of challenges concerning the establishment and maintenance of a stable therapeutic relationship and the development of the analytic process. For both, the experience of the virtual encounter, with its complexity of closeness and distance, presence and absence, played a major role, as well as the strong changes or limitations of sensory perception. We assume that observations from our study can be generalized to other psychodynamic methods and even other therapeutic orientations. Overall, it became clear that we still need to gain a much more profound understanding of the processes and dynamics of remote psychotherapy by applying both quantitative and qualitative research methods. Given the important role of remote psychotherapy for coping with the current crisis (Swartz, 2020), we believe that it is necessary to keep an open mind about new treatment formats without abandoning a critical stance.

## Data Availability Statement

The raw data supporting the conclusions of this article will be made available by the authors upon reasonable request for research purposes after signing a confidentiality and use agreement.

## Ethics Statement

The studies involving human participants were reviewed and approved by the Ethics Committee of the Austrian Society for applied Depth Psychology and Psychotherapy (ÖGATAP). The patients/participants provided their written informed consent to participate in this study.

## Author Contributions

AJ, BL, and JM: conceptualization and methodology. AJ and BL: qualitative data analysis. AJ: data curation, writing—original draft preparation, and project administration. JM and BL: writing—review and editing. All authors have read and agreed to the published version of the manuscript.

## Conflict of Interest

The authors declare that the research was conducted in the absence of any commercial or financial relationships that could be construed as a potential conflict of interest.

## Publisher’s Note

All claims expressed in this article are solely those of the authors and do not necessarily represent those of their affiliated organizations, or those of the publisher, the editors and the reviewers. Any product that may be evaluated in this article, or claim that may be made by its manufacturer, is not guaranteed or endorsed by the publisher.
